# A Novel Treatment for Neurotrophic Corneal Ulcer Using Topical Cenegermin (OXERVATE™) Containing Recombinant Human Nerve Growth Factor

**DOI:** 10.7759/cureus.11724

**Published:** 2020-11-27

**Authors:** Abhimanyu S Ahuja, Frank W Bowden, Jerry L Robben

**Affiliations:** 1 Surgery, Charles E. Schmidt College of Medicine Florida Atlantic University, Boca Raton, USA; 2 Ophthalmology, Bowden Eye & Associates, Jacksonville, USA

**Keywords:** cenegermin, oxervate, cornea, corneal ulcer, prokera, tarsorrhaphy, amniotic membrane transplantation, epithelial defect, neurotrophic keratitis, neurotrophic keratopathy

## Abstract

Neurotrophic keratitis represents a complex degeneration of the cornea that can result in debilitating symptoms and serious sight-threatening complications. Patients with neurotrophic keratitis (NK) usually present with a history of corneal injury or past surgical intervention involving damage to the corneal nerves; as well as, chronic ocular surface disease. This nerve damage results in sensory loss and poor healing to the corneal tissues. This poor ability to heal results in the downward spiral of events that NK patients are subject to, which can include corneal surface breakdown, ulceration, melting, and total perforation. Treatments to attempt to support the cornea to heal and then protect it have recently advanced beyond traditional treatments and may include amniotic membrane application, autologous serum eye drops, biologic eye drops, and various other supportive treatments. Even aggressive combinations of these, and many other treatments, can leave NK patients still uncontrolled and progressing to end-stage disease that would require invasive intervention resulting in an even worse prognosis. This case report describes a patient who demonstrated all three stages of NK. Multiple interventions were initiated, including tarsorrhaphy, autologous serum eye drops, Prokera amniotic membrane, antibiotics, bandage contact lenses (BCL) therapy, and PKP. However, the patient experienced variable and transient improvement with relapse within a few weeks with recurrent and persistent epithelial erosion and stromal ulceration. A novel eye drop, cenegermin (OXERVATE^TM^), an ophthalmic solution containing 20 μg/mL of a recombinant form of human nerve growth factor (NGF), was added to her treatment regimen allowing for successful management of NK and restoration of corneal integrity.

## Introduction

Neurotrophic keratitis (NK) is a rare corneal condition caused by damage of trigeminal innervation, leading to an impairment in sensory and trophic function with a subsequent breakdown of the corneal epithelium [1,2]. The pathological changes of NK are seen because impairment of corneal sensitivity interrupts trophic relations, which are essential for corneal physiologic renewal and wound healing [2]. It has been stated that in the setting of reduced or altered corneal trophic function and sensitivity, an epithelial defect that does not heal or heals and repeatedly breaks down, is clinically indicative of NK [1]. Several conditions that impair the trigeminal nerve can cause NK, such as herpetic keratitis, multiple sclerosis, diabetes, contact lens misuse, ocular chemical burns, corneal surgery, and cranial neurosurgery [1,2]. NK is classified into three overlapping stages based on the depth of corneal involvement or severity [3]. Stage 1 is characterized by corneal epithelial alterations, stage 2 is characterized by corneal erosion, and stage 3 is characterized by corneal ulceration [3]. Management of NK is stage-dependent and can be categorized as medical management, non-surgical intervention, and surgical management [3].

In 2018, cenegermin eye drops (OXERVATE^TM^), an ophthalmic solution containing 20 μg/mL of a recombinant form of human nerve growth factor (NGF), were approved for the treatment of NK in the United States [3,4]. Cenegermin is the first and only drug that has received approval for the treatment of NK [4]. NGF is a neurotrophin that supports corneal integrity and promotes restoration of corneal nerve function after injury [4]. Furthermore, NGF is known to promote corneal healing, maintain corneal epithelial stem cells, promote tear production, and induce epithelial cell proliferation and differentiation [4]. Phase 2 clinical trials, conducted on patients with moderate or severe NK, showed treatment with cenegermin (as compared to treatment with a vehicle) is associated with lower rates of disease progression and higher rates of complete corneal healing [4]. In this paper, we describe the effective utilization of recombinant human nerve growth factor in the management of NK associated with a therapeutic keratoplasty.

## Case presentation

An 84-year-old Caucasian female presented with a history of type 2 diabetes, cardiovascular disease with heart valve placement, chronic dry eye disease, and pseudophakia with YAG laser capsulotomy in both eyes. She was referred by another ophthalmologist for infectious keratitis due to methicillin-resistant Staphylococcus aureus (MRSA) in the left eye that poorly responding to treatment. She had been managed with a variety of topical medications (tobramycin, amphotericin B, moxifloxacin, and voriconazole). The patient reported that the corneal infection, OS, began following YAG laser capsulotomy in the left eye. She noted progressive visual blurriness, excessive dry eye awareness as well as mild scratchiness, and light sensitivity in the left eye.

On our examination, her BCVA was 20/25, OD, and 20/70, OS. Intraocular pressure (IOP) was 14 mmHg in the right eye and 12 mmHg in the left eye. Slit-lamp findings, OD demonstrated signs of meibomian gland dysfunction (MGD) of the lids with a clear cornea and a posterior chamber IOL with an open capsule. The OS demonstrated for MGD findings, 1+ conjunctival injection, severe corneal thinning with a 0.5 mm infiltrate inferiorly with confluent SPK, and superficial vascularization. Incomplete lid closure with blinking was noted, OU. Fundus examination revealed mild dot-blot hemorrhages in the macula with vessel attenuation, OU.

Diagnostic testing revealed abnormal MMP-9 testing (InflammaDry, Quidel, USA) and abnormal meibography (LipiView II, J&J Vision, USA) with moderate gland truncation and atrophy, OU.

The patient was advised that the corneal ulceration was likely the result of long term ocular surface disease exacerbated by the chronic exposure associated with her incomplete blinking. Infectious keratitis resulted from an epithelial disruption in the presence of MRSA. The inflamed ocular surface contributed to poor healing response despite conventional measures in progress.

The initial plan of treatment was to apply a tobramycin-soaked collagen shield with patch occlusion for two days followed by a Prokera AMT device (Bio-Tissue, USA). Topical antibiotics in progress were continued. Dry eye/MGD therapy was initiated, OU, which includes Xiidra (Novartis), hourly Oasis Tears Plus, warm compresses with the Bruder mask, and lid scrubs with Ocusoft foam. She also began oral HydroEye (ScienceBased Heath) vitamins and doxycycline, 50 mg once daily. The patient was advised at this visit that she may need a future corneal transplant if the ulcer does not heal.

Two days later the patient presented for Prokera placement minor procedure, OS, which was placed in the left eye with no complications. The patient was advised to continue her medications as prescribed but to discontinue patching.

Five days later, the Prokera membrane was disintegrated, revealing a further corneal stromal thinning with a 1.5 mm descemetocele. Plans were made to pursue a therapeutic penetrating keratoplasty (PKP), in order to avoid corneal perforation. The uneventful PKP was performed. She was managed with topical moxifloxacin, Durezol (Novartis), and Bromsite (Sun Pharma), along with autologous serum tears.

At two weeks later, she presented with pain, discharge, and declined vision, OS. The corneal graft showed edema with an inferior infiltrate with 4+ cell and flare with a 2 mm hypopyon. Corneal scrapings for cultures were obtained. The patient was started on fortified tobramycin and fortified Vancomycin hourly. A collagen shield was inserted. Durezol therapy was reduced, while moxifloxacin and Bromsite were stopped. Two days later, the cultures revealed MRSA that was sensitive to vancomycin. Tobramycin was discontinued. A collagen shield soaked in vancomycin was inserted with patch occlusion. Two days later the cornea graft showed a 5 mm x 8.2 mm epithelial defect with central stromal opacity, the hypopyon was reduced to 1/2 mm. Prior medical history demonstrated nasal MRSA colonization. She was advised that she would require another therapeutic PKP. Consultation with an infectious disease specialist for systemic treatment of the MRSA was arranged prior to surgery. Bactroban was recommended.

A second therapeutic PKP with lateral tarsorrhaphy was successfully completed, OS. The fortified vancomycin was discontinued. Moxifloxacin, Polytrim, and autologous serum were started with topical steroids and lubricants and full-time patch occlusion. At nine days later, an epithelial defect persisted at 40% of the graft surface. A bandage contact lens was inserted.

Over the next four weeks, vigorous medical management with lubricants, multiple Prokera devices, serial bandage contact lenses, and autologous serum were utilized. The epithelial defect persisted with corneal haze and little ocular discomfort. The vision was reduced to counting fingers. She demonstrated a clinical pattern consistent with neurotrophic keratitis. A decision was made to add a novel treatment cenegermin (OXERVATE^TM^) which is a recombinant human nerve growth factor. Topical Oxervate^TM^ was initiated six times daily.

Within five weeks after starting the cenegermin the patient’s vision and epithelial defect began to improve. After eight weeks of continued Oxervate^TM^ therapy, the epithelial defect had totally resolved and the cenegermin was reduced to QID while lubricants, steroids, and dry eye therapy were continued. Since this episode, the patient has required frequent observation. She maintains the lateral tarsorrhaphy, OS with the weekly replacement of bandage contact lenses and continued autologous serum therapy. She also maintains lid hygiene and dry eye measures to maintain her ocular surface, OU. Her quality of life has much improved and her vision has improved to 20/70 uncorrected in the left eye.

## Discussion

The patient experienced a problematic clinical course of infectious keratitis in the presence of corneal exposure, ocular surface disease, MRSA colonization, and neurotrophic keratitis. Persistent keratitis, diabetes, chronic ocular surface disease, and previous ocular surgery were contributing factors to the patient’s development of NK [2]. Multiple interventions were initiated, including tarsorrhaphy, autologous serum eye drops, Prokera amniotic membrane, antibiotics, bandage contact lenses (BCL) therapy, and PKP. The patient experienced variable and transient improvement with relapse within a few weeks with recurrent and persistent epithelial erosion and stromal ulceration. This patient demonstrated all three stages of NK [2].

Tarsorrhaphy is a very effective procedure in patients with NK, as it reduces corneal surface exposure which promotes epithelial healing [2,5]. However, despite, lateral tarsorrhaphy in the patient’s left eye, a recurrent corneal ulcer occurred in the medial area of the exposed corneal surface. Evidence has shown that amniotic membrane transplantation can successfully treat the persistent epithelial defects and corneal ulceration associated with NK [2]. In this patient, multiple Prokera amniotic membrane devices were utilized with limited benefit. This case demonstrates successful management of NK associated with a complex presentation of corneal exposure and penetrating keratoplasty, wherein Oxervate^TM^ therapy was added to conventional treatment measures.

It is thought that as a recombinant form of human NGF, cenegermin can aid the restoration of corneal integrity in patients with NK [4]. Two prior phase II randomized controlled trials (RCTs), which offer substantial evidence [6], showed cenegermin was more effective than a vehicle (considered a proxy for artificial tears) in improving corneal epithelial healing after eight weeks of treatment [7]. However, there is no published evidence supporting the clinical effectiveness of cenegermin versus other treatments used to manage moderate or severe NK [7]. 

## Conclusions

Our case highlights the effectiveness of the utilization of Oxervate^TM ^in the management of NK. Further studies are warranted to establish the efficacy of cenegermin added to conventional treatments for NK in the complicated corneal graft patient.
